# Sugar intake and all-cause mortality—differences between sugar-sweetened beverages, artificially sweetened beverages, and pure fruit juices

**DOI:** 10.1186/s12916-020-01579-w

**Published:** 2020-04-22

**Authors:** Floor R. Scheffers, Jolanda M. A. Boer

**Affiliations:** 1 Center for Nutrition, Prevention, and Health Services, National Institute for Public Health and the Environment (RIVM), PO Box 1, 3720 BA Bilthoven, The Netherlands; 2 Julius Center for Health Sciences and Primary Care, University Medical Center Utrecht, Utrecht University, Utrecht, The Netherlands; 3 Faculty of Health, Nutrition and Sport, The Hague University of Applied Sciences, The Hague, The Netherlands

**Keywords:** Sugar-sweetened beverages, Artificially sweetened beverages, Pure fruit juice, Mortality, Sugar, Dietary guidelines

## Background

In 2015, the World Health Organization (WHO) published the guideline “Sugars intake for adults and children” [1]. In this guideline, the WHO recommends a daily free sugar intake of less than 10% of total energy intake in both adults and children in order to reduce noncommunicable diseases (NCDs), with a particular focus on the prevention and control of unhealthy weight gain and dental caries. Free sugars refer both to added sugars and sugars naturally present in, for example, pure fruit juice, but not to sugars in intact fruits. Based on this guideline, a few countries consider pure fruit juice (without any added ingredients) nutritionally similar to sugar-sweetened beverages (SSBs), such as sodas with added sugars, in their dietary guidelines. However, most countries state in their dietary guidelines that pure fruit juice can (partially) replace whole fruit [2]. Artificially sweetened beverages (ASBs), which contain no or less sugars or calories, are not included in dietary guidelines worldwide. Current scientific evidence is mainly focused on SSBs and their health effects, while studies on pure fruit juices and ASBs are more scarce and inconsistent. The conflicting dietary guidelines for pure fruit juice and the absence of dietary guidelines for ASBs emphasize the need for more research on the health effect of these types of beverages.

## The UK Biobank study

The paper presented by Anderson et al. [3] studied whether SSBs, ASBs, and pure fruit juice consumption were associated with all-cause mortality in a very large prospective population-based cohort study. An important strength of this study is the distinction between SSBs, ASBs, and pure fruit juices. Many studies combine these different types of beverages and thereby mask possible differences. Moreover, specifically for fruit juice, many studies do not distinguish between pure fruit juice and sweetened fruit juice, and therefore, also no distinction can be made between added sugars and natural sugars in fruit juices. This study showed that consumption of SSBs was associated with all-cause mortality independent of total sugar intake and total energy intake, whereas pure fruit juice consumption was not associated with all-cause mortality. This finding implies different health effects for the consumption of pure fruit juice and SSBs. The fact that SSBs contain added sugars and pure fruit juice contains natural sugars could be one of the possible explanations for the different associations with all-cause mortality. Furthermore, in contrast to SSBs, pure fruit juice contains polyphenols which are associated with a lower all-cause mortality risk [4]. ASBs also initially appeared to be associated with all-cause mortality in the highest intake category (more than two ASBs per day). However, sensitivity analyses suggested reverse causation and residual confounding.

## Conclusions

While still more research is needed on the association of both pure fruit juice and ASB consumption with all-cause mortality risk, Anderson et al. provide an important contribution to the current scientific evidence in this field by finding different associations for all-cause mortality between consumption of SSBs and consumption of pure fruit juice. Since the association between SSB consumption and all-cause mortality is well established [3, 5, 6], it is very important to include the advice to reduce the consumption of SSBs in dietary guidelines. However, there is too little evidence to assume that pure fruit juice and SSBs are nutritionally similar and have the same health effects. Nevertheless, this does also not imply that pure fruit juice should be encouraged as a substitute for SSBs, since it still contains comparable amounts of sugar. As recommended by the WHO [1], drinking water instead of SSBs to reduce sugar intake would be the best advice. However, the current state of evidence does not make it possible yet to draw up dietary guidelines for ASBs.

## Data Availability

Not applicable

## Abbreviations

ASBs: Artificially sweetened beverages
NCDs: Noncommunicable diseases
SSBs: Sugar-sweetened beverages
WHO: The World Health Organization
